# “How Do Municipal Managers Describe Improving the Services Provided to Drug Death-Bereaved Persons?” a Qualitative Study

**DOI:** 10.1177/00302228231178870

**Published:** 2023-05-30

**Authors:** Hilde-Margit Løseth, Kari Dyregrov, Lillian Bruland Selseng, Sonja Mellingen

**Affiliations:** 1 Department of Welfare and Participation, Faculty of Health and Social Sciences, 1657 Western Norway University of Applied Sciences, Bergen, Norway

**Keywords:** drug-related death, bereavement, unnatural deaths, service delivery, managers, caregivers, qualitative

## Abstract

Bereavement following drug-related losses is potentially traumatizing and may cause adverse health outcomes. These bereaved experiences are unacknowledged and omitted in municipal health and welfare service delivery. Reflexive thematic analysis was applied to six focus group interviews with 26 municipal managers. Knowledge about municipal managers' perspectives for improving psychosocial follow-up to drug-death-bereaved persons was perceived as vital for improving the quality of public services. The findings show how the services are perceived to be affected by macro-, meso, and micro-level processes. The participants suggested that service delivery should be based on an integrated organizational approach. Political, organizational, administrative, financial, and communicative processes are addressed and discussed in light of Osborne’s theory of public service logic. The managers argue that a broad, contextual frame and infrastructure, enhancing managers' latitude of action regarding employees and facilitating collaborative service flexibility, would provide more sustainable, competent services.

## Introduction

Due to the high mortality and demanding social, financial, and individual circumstances in large populations, deaths related to illicit drug use are considered a public health problem (United Nations Office on Drugs and Crime, 2022; World Health Organisation, 2019). Recognizing the societal and individual severity of drug-related deaths (DRDs), national strategies for reducing fatalities have been implemented internationally (European Monitoring Centre for Drugs and Drug Addiction, 2021; World Health Organisation, 2019). Previous research has shown that many people bereaved by DRDs experience a lacking, confusing, or unkind service system (Dyregrov & Selseng, 2022; Lambert et al., 2021 pp. 144–151; Templeton et al., 2016; Titlestad et al., 2019; Van Boekel et al., 2013). This article explores how municipal managers describe suggested improvements in the services offered to DRD-bereaved persons. The findings are discussed with reference to Osborne’s theory of public service logic Ng & Vargo, 2018; Vargo & Lusch (2004) cited in Osborne, 2020 pp. 31–34; Rittel & Webber, 1973 p.162.

The USA Centers for Disease Control and Prevention reported 50,943 overdoses in 2021, a mortality rate of 28.3 per 100,000 (CDC, 2021). There were at least 5800 overdose deaths in the EU in 2020 (mortality rate: 17.4) (EMCDDA, 2022). In Norway, there were 324 overdoses in 2020 (mortality rate: 6.2) and 241 in 2021 (mortality rate: 4.5). DRDs encompass fatalities where drugs are the starting factor in a chain of events resulting in death or are a secondary, contributory cause (Amundsen, 2015 p. 17–19).

According to many people bereaved by DRDs, such losses are perceived as sudden and unnatural, leaving the bereaved traumatized and under great strain (Feigelman et al., 2020; Titlestad et al., 2019) (null). This is supported by research showing that sudden, unexpected, or unnatural deaths may be followed by shock and crisis reactions and are potentially traumatizing (Bottomley & Smigelsky, 2022). Those bereaved by DRDs are at a higher risk of complicated grief and health problems than those bereaved by other sudden deaths (Christiansen et al., 2020; Djelantik et al., 2020; Feigelman et al., 2011; Ford et al., 2018; Li et al., 2005; Titlestad & Dyregrov, 2022). The impact of a relative’s or close friend’s addiction to illicit drugs before a DRD may influence the grieving process, e.g., due to a high-risk and marginalized lifestyle, societal stigma, and challenging relationships. The bereaved individual experiences outsiderness and discriminatory treatment (Dyregrov & Selseng, 2022; Lambert et al., 2021, pp. 144–151; Templeton et al., 2016; Titlestad et al., 2019; Van Boekel et al., 2013). The affiliation with illicit drug use by the deceased, the associated social stigma, and the complexity of the current health and welfare services are contributing factors. Most DRD-bereaved individuals experience a lack of recognition and convey a need for more competence regarding drug-related deaths and bereavement in the service system (Kalsås et al., 2022; Lindeman et al., 2022, pp. 156–159; Templeton et al., 2016; Titlestad et al., 2019; Walter et al., 2017). The professional helper’s experiences and perspectives relating to meeting those bereaved by fatalities due to illicit drug use have been sparsely researched but may help to nuance this disheartening situation (Reime et al., 2022; Valentine, 2017 pp. 1–3) found that professional helpers admit unintentionally having neglected this group as potentially needing psychosocial follow-up. Valentine et al. (2018, pp. 298–299, 300–301 and Løseth et al., 2022) noted a complex service context, suggesting that the organizational and administrative systems may influence follow-up for both the bereaved and the practitioners. The main barriers are associated with the organizational and administrative complexity, the multitude of actors, and a lack of knowledge regarding the particular context of the stigma associated with illicit drug users (Lambert et al., 2021; Løseth et al., 2022; Valentine & Bauld, 2016; Valentine et al., 2018).

Although several barriers have been pointed out, we lack knowledge regarding how services can be improved. This paper aims to reduce this knowledge gap by exploring how Norwegian municipal managers describe what they perceive as necessary improvements in services relating to DRDs. The study presented in this article is based on the research question: “How do municipal managers describe improving the services provided to drug death-bereaved persons?”

Ideally, health and welfare services should make a positive difference in their recipient’s life conditions. Osborne et al. (2021 pp. 668–671) provide a framework that validates the complexity and interconnections in public services, offering alternative concepts of how the service deliveries are integrated, interact, and mutually influence each other by shifting from linear manufacturing and product-oriented logic to a more dynamic service-dominant approach. This article uses the framework to shed light on how municipal managers describe services on the macro- (institutional) level, the meso-level, which is related to the service delivery system and collaborative governance, and the micro-level, which comprises the individual service providers, users, and stakeholders. The framework emphasizes how practice on the micro-level cannot be separated from the macro- and meso-levels. Osborne et al., 2021 highlight the need for practitioners to work across the three ecosystem levels and be aware of how they can best influence value creation at different stages (European Monitoring Centre for Drugs and Drug Addiction EMCDDA, 2021).

### The Norwegian Context

As a welfare state, Norway has universal health and social services rights. The Public Health Act (PHA) aims to contribute to social development that promotes public health and equalize social health differences (PHA, 2012; § 1). Municipalities have obligations to plan, evaluate and correct their services to comply with the requirements described in laws and regulations (PHA, 2012 § 3–1, The Health Personnel Act, 1999 §§ 1–17, 35–38). Managers on executive and administrative levels have some autonomy but must maneuver between politics and administrative tasks. The managers holding positions in the context of practice service provision have personnel responsibilities, as well as professional social, political, and administrative responsibilities. Lack of financial and administrative support may hinder optimal professional solutions (Christensen & Legreid, 1998; null, pp. 20–24).

In the event of sudden or unexpected deaths, the obligation to provide proactive, comprehensive, and coordinated assistance to those bereaved by close friends or family is outlined in the national guidelines “Psychosocial interventions in the event of crisis, accidents and disasters” (Norwegian Directorate of Health, 2016 pp. 18–19). The guidelines underline the municipalities’ right to organize their psychosocial follow-up in the best way they see fit, as long as the services are justifiable. They also emphasize the obligation for all actors to cooperate across services (Norwegian Directorate of Health, 2016, p.18). According to the principle of collective effort, crisis teams are mainly assembled by employees from different organizational and administrative service levels and jurisdictions.

Managers on executive and administrative levels have some autonomy but must maneuver between politics and administrative tasks. The managers holding positions in the context of practice service provision have personnel responsibilities, as well as professional social, political, and administrative responsibilities. Lack of financial and administrative support may hinder optimal professional solutions (Christensen & Legreid, 1998; Vabø and Vabo, 2014, pp. 20–24).

## Method

### Context of the Study

This article is part of the END project (https://www.hvl.no/en/research/group/mental-health-and-substance-abuse/the-end-project/). The primary objective of the END project is to improve the life situation of persons bereaved following DRDs, exploring their experiences with and the service support they receive from the Norwegian municipal health and welfare services. The END project includes four studies with separate data collection procedures and research questions. Whereas three focus on the bereaved, the fourth is based on the helper’s perspective. This sub-study is grounded on focus group interviews with municipal managers and their descriptions of suggested improvements to the services offered to the DRD-bereaved.

### Design

The article’s empirical and analytical foundation consists of focus group interviews and demographic data collected in questionnaires. Using purposeful sampling and reflective thematic analysis, we have applied a flexible, inductive, and empirically driven approach to explore the municipal managers’ descriptions of suggested improvements to the health and welfare services (Braun & Clarke, 2019a). Interaction among the participants in focus groups can promote synergy and spontaneity and facilitates discussing and reflecting on opinions and experiences (Malterud, 2012, p. 18; Willig, 2013, pp. 30–31).

### Recruitment, Sample, and Participants

From fall 2017 to spring 2020, the participants were recruited in conjunction with The Norwegian Directorate of Health’s pilot project for Norwegian municipalities with a high incidence of overdose deaths. Information letters were sent to networks, medical officers, executives, managers, and clinical practice professionals. The project was announced via media, e.g., Facebook and radio. A “snowball recruitment” process followed through existing participants.

The criterion for recruitment to the helper’s sub-study was that the professional helpers should work in a position relating to the services provided for the bereaved following a DRD through the various municipalities’ health and welfare services and non-governmental organizations. The total sample of the helper study consisted of 105 professional helpers in 24 focus groups. The sample for this article derives from six focus group interviews with municipal managers, comprising 26 participants from various positions across the municipal leadership levels, such as superior staff positions close to the political administration (the chief medical officer, department managers, advisors, and qualitative coordinator), and various practitioners with leadership positions and managerial responsibilities from emergency and outpatient rooms, mental health, and addiction services (Table 1). The participants represented small and large municipalities. Four focus groups each had four participants, and two had five.

**Table 1.** Characteristics of the Interviewees in the Focus Groups (*N* = 26).

Position	Number
Department manager	5
Division manager	12
Professional and quality coordinator	1
Advisor	3
Municipal chief medical officer	1
Emergency nurse	1
Team manager of psychosocial crisis follow-up	2
Low-threshold outpatient field nurse	1

### Focus Group Interviews

Three separate teams conducted the focus group interviews, each consisting of an experienced senior scientist as the moderator and an assistant co-leader. The interview guide focused on the service areas, positions, and roles. The participants were asked to give examples of conditions in the organization that they thought were successful in providing good-quality services to the bereaved and what they perceived as essential factors in these situations. Other themes concerned challenging conditions in the organization of service delivery to the DRD-bereaved and how the services could be better organized. In addition, the helpers were questioned about their views on the proactive approach, how to improve initiatives for contacting the bereaved, and the necessary conditions for promoting service collaboration. Finally, they were asked what needs they perceived employees working with the DRD-bereaved have and what they could do to facilitate the psychosocial follow-up positively.

To ensure the best data co-creation, the teams prepared by discussing how to best leverage the focus groups as well as the approaches to communication. To yield the most profound insights, they discussed balancing openness and structure and addressing interaction and dynamics among the group members (Morgan, 2010; Tausch & Menold, 2016). A procedure guide was created to ensure that all interviews were consistent with the information provided to the groups. Each interview team practiced their roles and the use of the technical equipment beforehand. The interviews took place in or near the managers’ workplaces. All potential participants were given an informed consent form stating that participation was entirely voluntary and consisted of one focus group interview of approximately 2.5 hours in duration, as well as a questionnaire on background variables. The interviewers immediately summarized their impressions in writing after the interviews. The interviews were audiotaped and transcribed verbatim by a professional transcriber, consisting of 142 pages of text.

### Analytical Approach

Due to the sparse knowledge of professional services offered to the DRD-bereaved, an inductive, flexible, and empirically driven approach was employed to explore the interview data (Braun & Clarke, 2014). Braun and Clarke (2019a) describe six phases in the reflexive process where each phase builds on the previous one, and the method emphasizes the interaction among the participants. An inductive bottom-up approach was applied to the data set, in which themes were searched to gain an overall impression and identify meaning patterns. The analysis and coding took place simultaneously and repeatedly. The first author read the transcriptions several times, noted initial thoughts on themes, codes, and meanings units, and created a preliminary table to maintain oversight. The researchers did not engage with theoretical literature in the first stages of this inductive approach. The author team reviewed the suggested themes and codes several times and finally agreed on two themes. In line with Braun and Clarke’s (2019b) recommendations, the authors reflected on their contributions to the reflective process in both the interviews and the analytical process. The topics discussed were the variety in the interview teams, the researchers’ professional backgrounds and experiences, and the assumptions and actions in the interview processes. The authors’ occupational backgrounds include a psychiatric nurse with an MA, and a psychologist, a social worker, and a sociologist with PhDs. They have long-term professional experience in health and welfare services and research on crisis and traumatic grief, addiction, and mental health. The authors’ backgrounds were significant in understanding the dynamics and statements in the group interviews and the interpretation during the analytical process.

### Findings

**Figure 1.**
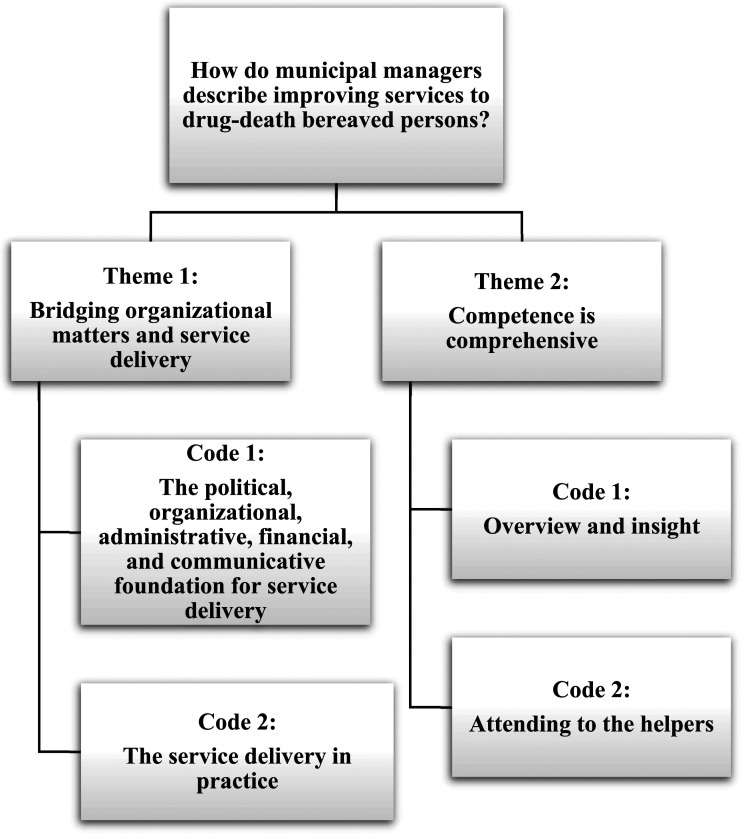
Municipal managers’ descriptions of improvements to services delivered to drug death-bereaved persons.

A clear pattern was that the participants realized that the DRD-bereaved was mainly overlooked as subjects of potentially traumatizing losses and that this lack of attention needed improvement. Several participants called for heightened political awareness of the DRD-bereaved, arguing that recourses would be available if the political focus were explicit. The participants suggested key areas where municipality services to DRD-bereaved persons could be improved, comprising a broad, contextual infrastructure and competence. The analysis identified two themes: (I) bridging organizational matters and service delivery, and (II) competence is comprehensive. (Figure 1)

### Theme 1: Bridging Organizational Matters and Service Delivery

The first theme comprised organizational structures between as well as within municipalities and other administrative levels. It included prerequisites affecting the managers’ and employees’ latitude of action. This theme was coded into a) the political, organizational, administrative, and financial foundation for actions and b) the service delivery in practice.

#### Code 1: The Political, Organizational, Administrative, and Financial Foundation for Actions

The majority of the participants were concerned with organizational matters and emphasized the importance of having an overview of existing services. An executive planner summed up the interconnection of needed improvements:

From a manager’s perspective, it (*leadership)* is very connected. It is about having a good competence and recruitment plan, which is about what basic competence you recruit; you must have good basic competence, so you must ensure that you get good employee training. You have to have good management, you have to have a good structure, you have to make sure that you have the routine… This has to work in an interaction, and that there is a culture of feedback, where employees stand out, who know what is not in place… that there are ways to convey it in the line, which means that we are constantly able to adjust ourselves. You must be able to adapt when new changes come, like requirements and guidelines. (ID39)

Most participants perceived that overall structures regarding the administrative and financial division of the services did not fit the assignment of psychosocial follow-up with the DRD-bereaved. Several pointed to a need for a distinct and coherent strategy across the departments to avoid tension in mid-level management about which services were responsible for allocating time and resources for employees to attend courses and training. A crisis team manager discussed how explicit political awareness improved service conditions by allowing the department and division managers to prioritize and schedule annual meetings with the involved actors (police, emergency services, and crisis teams) to provide psychosocial follow-up in the acute phase. The meetings were described as essential for increased assurance and trust and contributed to flexibility between actors with very different professional backgrounds and understandings.

The involved services had different affiliated financial regimes, which many participants found challenging. Most conveyed that the organizational principle of collective effort created barriers, e.g., a competitive mentality between divisions because those providing the most employees to crisis teams also had the most expenses. The willingness to cooperate between service levels was perceived as good, but they noted the absence of formal collaboration agreements between the services. Formal agreements were considered essential for clarifying services’ administrative and financial responsibility to the DRD-bereaved needing follow-up. Most participants wanted simpler systems, as illustrated by this executive administrator in a large municipality: “… there is performance-based funding in one place, framework funding in another, grant funds, municipal grant funds, state funds, and boxes like that. If I could have had one pot of money...” (ID42).

#### Code 2: The service Delivery in Practice

The participants in all focus groups acknowledged a need to organize the services to avoid continuity gaps, e.g., clinical pathways or designated persons/small teams attending to the bereaved. Despite having obligations to a psychosocial follow-up team in the job description and rota systems, several division and department managers perceived the collective effort as challenging because of the complexity of service delivery in psychosocial follow-up with those bereaved by DRDs. To ensure the best competence and personal and relational team fit, they wanted the local managers to have the latitude to maintain a close leadership, e.g., assemble the crisis teams.

In the focus groups from two larger cities, the participants problematized how the organizational complexity contributed to notifications of deaths not reaching adequate services. The information systems did not take into account lifestyle factors such as the deceased having several or no addresses, making it more challenging to inform the affected services and locate the bereaved. To remedy this, an executive planner suggested implementing easy-access solutions in the internal information systems, e.g., notes in journal systems that made information about a death apparent or flags implying that proactive psychosocial follow-up should be initiated (ID41).

Participants in all focus groups reported a lack of cooperative strategies and meeting points, causing communication gaps and consequential errors. However, one of the municipalities had scheduled four annual meetings between the crisis teams and the police, which were perceived as significantly increasing and improving communication. In cases of prolonged psychosocial follow-up, some division managers emphasized the importance of a relational approach and flexibility toward the DRD-bereaved being a vulnerable group. They arranged for the follow-up to continue with professionals from the first meeting, even if this meant crossing over departments. A local manager in a housing and rehabilitation center stated: *“*There shouldn’t be anything attitudinal that stands in the way of that, even if there are more practical reasons for it… but we are dependent on quite a good structure for it” (ID52).

### Theme 2: Competence is Comprehensive

The second theme included the overall understanding that competence should be considered at all levels and was coded into a) overview and insight and b) attending to the helpers.

#### Code 1: Overview and Insight

Most participants talked about a general lack of an organizational overview of the current services, and all participants emphasized the need for a general rise in competence in drug-related bereavement. All focus groups addressed the need for updated information on assigned responsibility areas, interfaces, and when, where, and how there should be collaboration. Although some municipalities had designated research and development departments or ongoing quality control and digital information development processes, several participants wanted more coherent organizational and administrative systems. They considered recruitment and competence plans for implementing new insights part of overarching strategies that should be included in the service’s annual management plans, including an embedded incident assessment on service delivery. It was suggested that unified competence programs grounded in agreements across administrative and service delivery boundaries should be included in all services providing employees to crisis teams.

A pattern in our findings was that the bereaved who used drugs were perceived as different from other DRD-bereaved and needed special arrangements. Thus, procedures for follow-up were not initiated because these deaths were mainly understood as an extension of the deceased’s drug use. However, few participants addressed this specific challenge on a concrete level. An exception was one advisor and strategic planner who pointed out that acute situations may be turbulent, so employees should have the opportunity to practice chaotic, potentially conflict-bearing situations, suggesting collaboration with university programs. She emphasized the need for competence in understanding the particular situation regarding illicit drug use and the stigma and shame many of these bereaved would have experienced:

There are perhaps three things in particular that you can add to the basic skills that the employee must have in bereavement work when it comes to this topic (those bereaved by DRDs). You need to have a little extra eye for the discrimination, the shame, and the ostracism these next of kin may have as an additional burden... It may be a family secret, and they have not told it to their friends; they have not wanted to tell it at the workplace, this secret and the shame and discrimination, and they may also experience this when they talk about it. Then they don’t meet understanding; they meet rejection. In some of these cases, there is crime, which affects the next of kin... It can be “torpedoes” that come, call and are unpleasant and they have to collect unpaid debts… The next of kin may also have been a victim of violence in close relationships, forced to sign as a guarantor, and robbed of many funds… And this duality then in the bereavement, to be relieved that he is dead, to mourn that he is dead… that is complicated. (ID30)

#### Code 2: Attending to the Helpers

Most participants perceived knowledge as essential in terms of general attitudes toward the DRD-bereaved, who already carried burdens of shame and loneliness in their grief. Several participants addressed the importance of being good role models as managers to reduce the stigma concerning illicit drug use. They were conscious of their positions and deliberately conveyed positive attitudes about collaboration to all parties involved in psychosocial follow-up after unnatural deaths. Educational programs within the services were considered vital to ensure good attitudes toward all involved stakeholders, illustrated by this division manager for addiction services: “The user group we are talking about is completely forgotten… in a way, they *(people*) don’t want to hear anything about it. I think it is vital that we are good role models and inform and create good attitudes” (ID40).

Several participants discussed how working with the bereaved experiencing shock and crises is demanding work, both professionally and personally. They emphasized that these employees need solid bereavement, crisis, and trauma knowledge, as well as relational competence, empathy, and a self-reflective capacity. However, our participants expressed split views on what they considered the best competence foundation overall and what should be the expert knowledge possessed by designated services. For example, some viewed crisis competence as an expert field of its own, while some regarded this as part of the professional’s basic competence. Others called for special teams with expert knowledge in bereavement, i.e., regarding DRDs. Most of these particular participants called for enhanced grief competence and skills training, which were assessed as necessary for improving and maintaining the employee’s competence in a neglected field.

Some participants described competence in the relational perspective, as well as understanding personal attitudes and reflective communication skills, as crucial for ensuring good-quality services and a sustainable workforce. Due to collective effort being the overall organizational principle for crisis teams, these managers expressed concern about how this organizational frame complicated attending to the crisis team members. For instance, the composition of a crisis team was considered an essential feature for good working relationships. The managers wanted the authority to assemble local crisis teams based on an overview of the various employees’ skills and personalities. To improve conditions for the existing crisis teams and recruit the best-suited employees, some managers had started advertising for employees especially interested in crisis work. They focused on the candidate’s suitability and interest for this type of professional work and their understanding of the relational conditions in play.

## Discussion

The findings from the municipal managers suggest that the organizational framework affects management, service delivery, and competence upkeep. Their suggestions recommend less complicated service delivery conditions and illustrate the interconnection between the macro-, meso- and micro-levels (Doberstein, 2016; Osborne et al., 2021, p. 837).

### Institutional level – Mirroring the values of the DRD-bereaved

According to Osborne et al. (2021), societal values and norms at the institutional level are mirrored in public services. Despite Norway’s universal rights for all citizens, previous research has shown that the services have neglected the bereaved from drug-related deaths (Løseth et al., 2022; Reime et al., 2022). Studies from Norway, the UK, and the USA have pointed out that those bereaved following DRDs are generally not acknowledged in the health and welfare services as bereaved by unnatural deaths (Løseth et al., 2022; McKell et al., 2018; Titlestad et al., 2019; Valentine et al., 2018; Walter et al., 2017). This corresponds with the findings from works focusing on the perspective of bereaved people (Feigelman et al., 2011; McKell et al., 2018; Orford et al., 2013; Templeton et al., 2017). These outcomes are also in accordance with several of the interviewed managers’ points. Many participants mentioned the attitude toward illicit drug users as a social problem and suggested, among other issues, increased knowledge as an area for improvement and a need for greater awareness of drug-related deaths and bereavement in society. In one focus group in particular, the participants discussed negative attitudes toward illicit drug users on a general societal level. They addressed the need for enhanced awareness of DRDs and bereavement among politicians, which corresponds with Osborne et al. (2021), who highlight the impact of societal norms, rules, and beliefs on public service delivery. Although the governmental values of equality and inclusion are outlined in the Norwegian legislation (Folkehelseloven [The Norwegian Public Health Act], 2012; Helse-og omsorgstjenesteloven [The Health and Care Services Act], 2012; Helsepersonelloven [The Act on healthcare personnel], 2001), the outcomes imply that a lack of knowledge and the societal stigma affiliated with illicit drug users have affected the professionals’ understandings. This detrimental situation could be perceived as drug-related losses reflecting a contextual and cultural value set regarding the marginalized position of illicit drug users and the associated stigma for their next of kin and bereaved (Doka, 2002; Dyregrov & Selseng, 2022; McKell et al., 2018; Templeton et al., 2017; Titlestad et al., 2019; Walter et al., 2017).

### Improvements Needed in the Service Delivery

According to Osborne (2021, p. 670), the meso-level relates to the processes concerning service delivery and the level the managers can most control. However, although they are key personnel in service provision, participants from various positions describe an organizational system they perceive as working against them. Most of the desired improvements are conveyed against the background of a system they find does not give them the tools necessary to provide the services fulfilling the assignment.

An unexpected death leaves many bereaved people in a state of shock and crisis and unable to make choices or attend a qualified assessment of the best service for them (Løseth et al., 2022; Stevenson, 2017; Winnicott, 1965). This specific knowledge of bereavement from sudden losses is the foundation of the national guidelines in Norway’s proactive approach (Norwegian Directorate of Health, 2016). To prevent adverse health outcomes, the national guidelines emphasize that those bereaved by unnatural deaths must receive early intervention through a proactive strategy by competent personnel combined in crisis teams (Health., 2016 pp. 14, 56). Unlike most municipal services, psychosocial follow-up is recommended as an outreach activity based on rapid notification from emergency services to the local crisis teams, which are competent to assess if follow-up should be initiated. The national guidelines emphasize mobilizing resources to be used flexibly and over time in collaboration between all actors, which corresponds with the participants’ suggestions for overall improvements.

Løseth et al. (2022) found that locating the bereaved and initiating psychosocial follow-up could be challenging due to the complex helping context, timeframe, and many actors involved. The findings in this article imply that crisis teams organized as an additional activity may add tension and entail the various actors becoming opponents, not co-players. Osborne’s approach validates the complexity between the different stakeholders and emphasizes the connection between government guidance, management, and service delivery, as well as the interplay between various levels and sectors, in addition to the broader societal context (Osborne, 2020; Osborne et al., 2021). However, the various parties represent different contexts and have different professional cultures and assignments. For example, in Norway, ambulance workers belong to the specialist health service, even for pre-hospital deaths. They are, therefore, subject to the affiliated legislation of this particular organizational level, whereas the police are organized as a separate government unit with its own directives. The municipality services operate within their regulations. The professionals have designated tasks and must prioritize (Løseth et al., 2022). Thus, when managers of local crisis teams consider the crisis team to be a distinctive service unit, they enter a complex system affecting the different legislation areas, service chains, and other service delivery managers, each with their assignments, mandates, interpretations, prioritizations, and logic (Helland, K.E., 2002). These issues are not necessarily solved but must be contextually understood and managed (Vabø & Vabo, 2014). Most participants in our analysis conveyed experiences describing how interrelationships and professional backgrounds between the various parties complicated their work. The managers in several focus groups noted a need for planned meeting points addressing communication, collaboration, and understanding of the various services positions and assignments. They also included responsibility boundaries, rota systems, and legislation as issues affecting the interrelationships and that such themes would have to be appropriately addressed in an overall plan. Two focus groups provided examples of how such planned meetings had improved communication and trust, increasing the flexibility between the services. Their suggestions represent examples of how managers can engage in service delivery, including making favorable conditions for transferring learning within the services.

### Competence

Competence was emphasized as an area encompassing the interconnections in psychosocial follow-up. The importance of education for all health and social personnel was perceived as essential for improving attitudes toward the DRD-bereaved, who already carry burdens of shame and loneliness in their grief. Many participants called for an overall rise in competence relating to bereavement regarding trauma and illicit drug use for all involved actors and emphasized that it should be a part of recruitment plans and integrated into the over-arching strategies to be coherent. Several managers also conveyed knowledge, understanding, and experiences of the emotional labor of attending to people in shock and crisis and addressed a need for closer relationships with their local crisis team members. They expressed concern for the well-being of these employees because they had little insight into how the local managers attended to the crisis team members. They called for a mutual understanding of the various service delivery areas that better facilitated the management of the crisis teams. In Osborne’s approach, the focus is shifted away from what the public service providers view as a measure of what constitutes a successful service. “Value” is emphasized here as the service’s purpose and is created by the service user’s engagement in the services and the interaction with professionals (Osborne, 2020, p. 2). Several managers pointed out that their competence to meet the bereaved significantly influences value co-creation. The knowledge of DRDs as special (Guy & Holloway, 2007), as well as the processes of associated stigma (Dyregrov & Selseng, 2022) and disenfranchised losses (Thompson & Doka, 2017 pp. 177–190), could enhance the competence of the managers and the service providers. Such knowledge could also offer concepts for communicating complicated issues for the employees and in meetings with the bereaved and, thus, engage and facilitate added value (Osborne et al., 2021, p. 670). Our findings show that the participants agreed with the requirements of the national guidelines (Norwegian Directorate of Health, 2016) and the governmental Coordination Reform (Meld St. 47 (2008-2009)), but were experiencing the overall separation of the organizational structures restraining them. Overall, the participants underlined the need for a competent overarching policy embedded throughout the service apparatus.

Competence in DRD and DRD bereavement was perceived as crucial for improving services. The municipal managers’ suggestions address contextual and practical conditions and emphasize the interconnections with competence, recruitment, and managerial authority in leadership. More research is needed to investigate an integrated organizational and administrative approach in municipal services to the psychosocial follow-up with bereaved individuals following drug-related deaths.

### Rigor and Trustworthiness

The investigation is embedded in the END project’s (https://www.hvl.no/en/research/group/mental-health-and-substance-abuse/the-end-project/)

Comprehensive data on drug death bereavement. Focus group interviews and reflective thematic analysis (Braun & Clarke, 2020) were conducted with participants from various municipal managerial positions to facilitate reflective processes as they could relate to each other from various positions. The cross-sectional grouping of managerial participants enhanced the participants’ awareness of drug-related deaths and bereavement. The recruitment procedure and analytical process have been validated by describing the method and the outcomes and adding quotations to improve the study’s transparency and validity. An experienced senior researcher conducted the interviews in every municipality and reflected on the interview processes with a less experienced co-moderator. In line with reflexive thematic analysis’s emphasis on reflexivity and subjectivity (Braun & Clarke, 2019b), the authors thoroughly discussed the data and findings in multiple sessions, ensuring the analytical trustworthiness by addressing the various managers’ positions, professional backgrounds, experiences, group dynamics, and interactions.

The weaknesses relate to focus group methodology in general during the interviews, e.g., relational and communication-related processes rooted in previous encounters. The data are derived from the participants’ descriptions of their experiences and assumptions, as well as the discussions and reflections in the focus groups. In general, purposeful sampling may lead to possible confounders in the chosen municipalities regarding geography, demographics, and exposure to drug-related deaths and bereavement.

## Conclusion

This study examined how municipal managers describe improving the services provided to DRD-bereaved persons. Managers in public services must balance governmental policies in public institutions where resources are scarce. Acknowledging the impact of societal values on the public services they manage is crucial for improving service delivery to a neglected population. The participants suggested that psychosocial follow-up with the bereaved following drug-related losses should be a prioritized political area. The findings show how the various organizational structures affect the management and operating of service delivery and the administration of competence upkeep. Integrating organizational and administrative processes with local managerial authority was perceived as providing better possibilities for purposeful recruitment and training, as well as improved employee care and utilization of resources, in addition to more sustainable, competent services.
